# TIR-NBS-LRR genes are rare in monocots: evidence from diverse monocot orders

**DOI:** 10.1186/1756-0500-2-197

**Published:** 2009-09-28

**Authors:** D Ellen K Tarr, Helen M Alexander

**Affiliations:** 1 Department of Ecology and Evolutionary Biology, University of Kansas 1200 Sunnyside Avenue, Lawrence, Kansas, USA; 2 Department of Microbiology and Immunology, Arizona College of Osteopathic Medicine, Midwestern University, 19555 North 59th Ave, Glendale, Arizona, USA

## Abstract

**Background:**

Plant resistance (*R*) gene products recognize pathogen effector molecules. Many *R *genes code for proteins containing nucleotide binding site (NBS) and C-terminal leucine-rich repeat (LRR) domains. NBS-LRR proteins can be divided into two groups, TIR-NBS-LRR and non-TIR-NBS-LRR, based on the structure of the N-terminal domain. Although both classes are clearly present in gymnosperms and eudicots, only non-TIR sequences have been found consistently in monocots. Since most studies in monocots have been limited to agriculturally important grasses, it is difficult to draw conclusions. The purpose of our study was to look for evidence of these sequences in additional monocot orders.

**Findings:**

Using degenerate PCR, we amplified NBS sequences from four monocot species (*C. blanda*, *D. marginata*, *S. trifasciata*, and *Spathiphyllum *sp.), a gymnosperm (*C. revoluta*) and a eudicot (*C. canephora*). We successfully amplified TIR-NBS-LRR sequences from dicot and gymnosperm DNA, but not from monocot DNA. Using databases, we obtained NBS sequences from additional monocots, magnoliids and basal angiosperms. TIR-type sequences were not present in monocot or magnoliid sequences, but were present in the basal angiosperms. Phylogenetic analysis supported a single TIR clade and multiple non-TIR clades.

**Conclusion:**

We were unable to find monocot TIR-NBS-LRR sequences by PCR amplification or database searches. In contrast to previous studies, our results represent five monocot orders (Poales, Zingiberales, Arecales, Asparagales, and Alismatales). Our results establish the presence of TIR-NBS-LRR sequences in basal angiosperms and suggest that although these sequences were present in early land plants, they have been reduced significantly in monocots and magnoliids.

## Background

Plants recognize pathogens using both non-specific and specific mechanisms. Pattern recognition receptors (PRRs) mediate non-specific recognition by interacting with microbe- or pathogen-associated molecular patterns (MAMPs or PAMPs), while the products of plant resistance (*R*) genes recognize specific pathogen molecules [1,2]. Disease resistance is the only known function for *R *genes, which appear to have a gene-for-gene relationship with pathogen avirulence (*avr*) genes [3].

**Figure 1:**
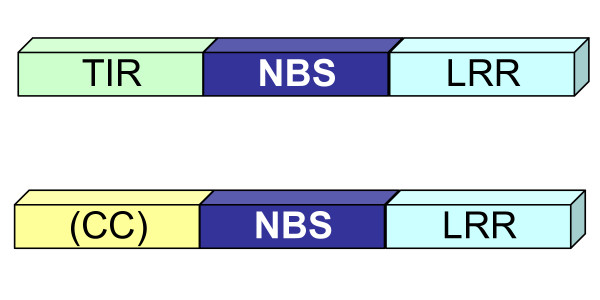
**Two types of plant NBS-LRR proteins**. The two classes of NBS-LRR protein are differentiated by the N-terminal domain. TIR-NBS-LRR proteins have a Toll-interleukin-like receptor (TIR) domain, based on homology to the *Drosophila *Toll and mammalian Interleukin-1 (IL-1) receptors. The N-terminal region of non-TIR-NBS-LRR proteins is less defined, but often contains a coiled-coil (CC) domain. In *R *genes, the NBS domain plays a role in intramolecular interactions with the LRR and N-terminal domains [28]. The N-terminal domain influences the signaling pathway that will be activated upon effector recognition [29], and may also be involved in pathogen recognition and interactions with targets of pathogen effectors [30].

Many *R *genes code for proteins containing nucleotide binding site (NBS) and C-terminal leucine-rich repeat (LRR) domains. The NBS domain of plant *R *genes (also called the NB-ARC domain) shares homology with human APAF-1 and *C. elegans *CED-4, proteins involved in regulating cell death [4]. NBS-LRR proteins can be divided into two groups, TIR-NBS-LRR and non-TIR-NBS-LRR, based on the structure of the N-terminal domain (Figure 1) [5,6].

The NBS domain from *R *genes is relatively conserved and contains type-specific motifs (Table 1). The final residue of the kinase-2 motif is especially useful for classifying a sequence as TIR or non-TIR [7]. TIR-type NBS sequences are relatively homogeneous and form a single clade, while non-TIR sequences form multiple clades that likely originated before the split between angiosperms and gymnosperms [8,9].

**Table 1:** Consensus motifs in TIR vs. non-TIR NBS sequences

**Gene Class**	**RNBS-A**	**Kinase-2**	**RNBS-D**
TIR-NBS-LRR	FLENIRExSKKHGLEHLQKKLLSKLL	LLVLDDV**D**	FLHIACFF

Non-TIR-NBS-LRR	FDLxAWVCVSQxF	LLVLDDV**W**	CFLYCALFPED

Consensus motifs are those reported by Meyers [7]. The final position of the kinase-2 domain that is used for classification is bolded and underlined.

The TIR class is found in bryophytes [10], and both TIR and non-TIR sequences are found in gymnosperms [11,12]. While both classes are present in eudicots, studies in monocots have reported only non-TIR sequences [7-9,13]. It is thought that TIR-NBS-LRRs either never developed in monocots [10] or have been lost [7-9,13]. However, four resistance gene analogs (RGAs) from the *Triticum*-*Thinopyrum *alien addition line TAi-27 have a kinase-2 motif consistent with TIR-NBS-LRR sequences [14].

**Figure 2:**
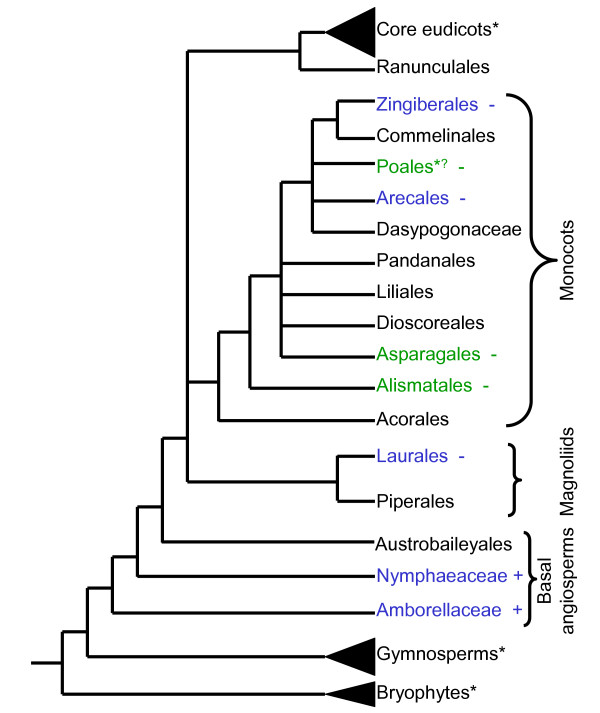
**Taxa included in this study**. The tree shows the ten orders and one family that form the monocots [19]. The broad relationships between the monocots and other land plants are shown. Groups marked with an asterisk (*) show where TIR-type NBS sequences have been confirmed. The status of TIR-type NBS sequences in Poales is unclear (*?) since these sequences are generally considered absent from Poales, but have been found in one study [14]. Monocot orders in green correspond to NBS sequences obtained in this study by degenerate PCR while those in blue show where sequences in this study were obtained from databases. TIR-type NBS sequences found or not found in this study: + or -

Studies of NBS-LRR sequences in monocots have been limited to agriculturally important species in the grass family (Poaceae). Recent studies from *Zingiber *and *Musa *species (order Zingiberales) reported only non-TIR type sequences [15-18]. Since there are ten orders of monocots [19], we are limited in our ability to make generalizations based on information from only two orders. To further investigate the presence of TIR-NBS-LRR sequences in monocots, we combined PCR and bioinformatics to obtain data from additional monocots as well as magnoliids and basal angiosperms (Figure 2).

## Results

We amplified sequences from four monocot species representing three monocot orders (Figure 2): *Draceana marginata *and *Sansevieria trifasciata *(Asparagales), *Spathiphyllum *sp. (Alismatales), and *Carex blanda *(Poales). For comparison, we included a gymnosperm (*Cycas revoluta*) and a dicot (*Coffea canephora*). We obtained sequences from a total of 60 PCR products that resulted in 24 unique NBS sequences (Table 2). We found non-TIR type sequences in all plants tested except the cycad, but only two unique TIR-type NBS sequences, one each from *C. revoluta *and *C. canephora*.

**Table 2:** NBS sequences obtained by PCR

**Species**	**Number of fragments successfully cloned and sequenced by primer specificity**			**Number of fragments that resulted in TIR vs. non-TIR NBS sequences**		**Number of unique NBS sequences obtained**
	**TIR primers**	**Non-TIR primers**	**General primers**	**TIR**	**Non-TIR**	

*Carex blanda*	11	5	8	0	3	5

*Cycas revoluta*	3	1	2	3	0	1

*Dracaena marginata*	2	1	5	0	3	3

*Sansevieria trifasciata*	8	1	1	0	2	2

*Spathiphyllum sp*.	1	1	4	0	4	9

*Coffea canephora*	2	0	4	1	3	4

*Arabidopsis thaliana	5	0	2	3	0	3

Total (excluding *A. thaliana*)	27	9	24	4	15	24

For each species tested, the table shows the number of fragments successfully cloned and sequenced for each type of primer set, the number of these fragments that produced TIR and non-TIR sequences, and the number of unique NBS sequences found. Based on previous work, we expected PCR products of approximately 700-900 base pairs [8,9]. In general, we cloned fragments of approximately 600-1000 bp, but we also cloned some fragments as small as 300 bp and as large as 1.5 kb to allow for the possibility that the NBS domain of TIR-type sequences in monocots differs significantly from those observed previously. At least five fragments smaller than expected and five larger than expected were cloned and sequenced, none of which contained identifiable NBS sequence. We cloned a total of 62 fragments and successfully obtained 105 sequences from 60 of those fragments. The BLASTP algorithm was used to compare the translations to the Genbank non-redundant database. A conserved domain search identified 30 sequences from 19 fragments that showed homology to an NB-ARC domain. We excluded six sequences that did not contain an open reading frame, were redundant, or were fragments identical to longer sequences, resulting in a total of 24 unique sequences.

* A. thaliana was used only to confirm that TIR-specific primers would amplify TIR-type NBS sequences. No non-TIR fragments were cloned from *A. thaliana*. The table only shows *A. thaliana *sequences obtained that included an open-reading frame from 5' to 3' primer. Additional sequences obtained that included introns were not included in the table.

Using Pfam [20] and GenBank [21], we retrieved 17 monocot sequences (ten from *Musa acuminata*, four from *Elaeis guineensis*, and three from *Zingiber *species), all of which we classified as non-TIR-NBS-LRR sequences based on the kinase-2 motif. Although we did not find any new TIR-type sequences from monocots, the search confirmed the similarity of the *Triticum*-*Thinopyrum *sequences [14].

In addition to monocot sequences, we retrieved two sequences from *Persea americana *(magnoliid) and seven sequences from basal angiosperms (five from *Nuphar advena *and two from *Amborella trichopoda*). Based on the kinase-2 motif, both *P. americana *sequences were non-TIR and all five *N. advena *sequences were TIR-type sequences. The *A. trichopoda *sequences have a glutamic acid in the diagnostic position, but downstream motifs similar to TIR-type NBS sequences (data not shown). For comparison, we also retrieved 37 *Pinus *(gymnosperm) and six *Physcomitrella patens *(bryophyte) sequences.

**Figure 3:**
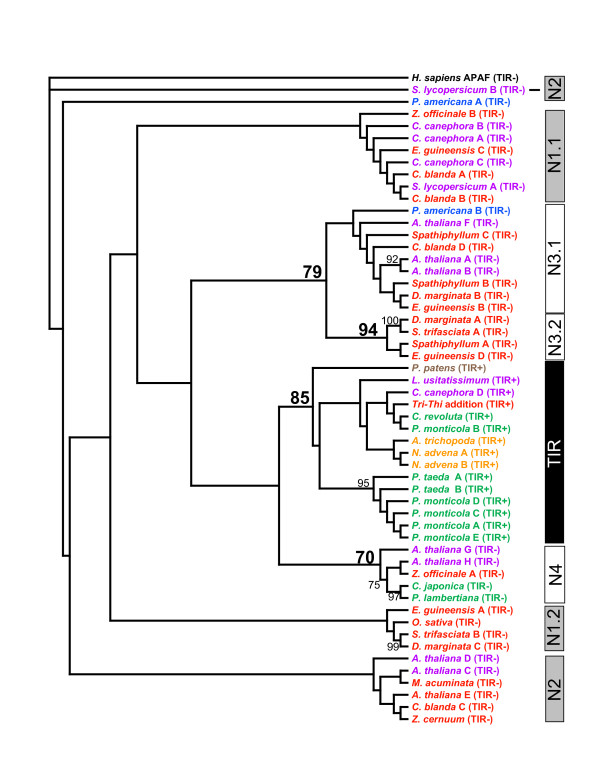
**Phylogenetic tree**. We performed a phylogenetic analysis of representative NBS sequences using parsimony criteria (heuristic searches, parsimony default parameters with 100 random sequence additions). The species of each sequence is shown with a letter designation (if more than one sequence from the species was used) and whether sequence analysis shows TIR (TIR+) or non-TIR (TIR-) sequence motifs. Monocot sequences are shown in red, eudicot sequences are shown in purple, magnoliid sequences are shown in blue, basal angiosperm sequences are shown in orange, gymnosperm sequences are shown in green, the bryophyte sequence is shown in brown, and the outgroup human sequence is shown in black. Bars on the right show a classification of NBS sequences modified from groups reported previously [8]. Numbers shown are from bootstrap analysis (1000 replicates) using parsimony criteria. Only values over 70 are shown.

We eliminated redundant sequences within a species (>70% identity), resulting in an analysis of 53 plant sequences (Table 3 and Additional Files 1, 2 &3) and human APAF-1 as an outgroup sequence. As much of the NBS domain as was available for each sequence was used for phylogenetic analysis using parsimony criteria (Figure 3). Based on the scaffold tree (see Methods), we compared our clades with those previously reported [8]. All fifteen sequences that we identified as TIR-type NBS sequences based on consensus motifs formed a single clade that was well-supported by bootstrap analysis (85%). The non-TIR-type NBS sequences formed several clades, but many were not well-supported. The well-supported non-TIR clades correspond to non-TIR clades 3 and 4 in Cannon's analysis [8], while non-TIR clades 1 and 2 are more ambiguous.

**Table 3:** Taxa included in phylogenetic analysis

**Species**	**Number used for tree**	**Pfam seed sequence**
*Arabidopsis thaliana*	8	6

*Amborella trichopoda*	1	

*Carex blanda*	4	

*Coffea canephora*	4	

*Cryptomeria japonica*	1	

*Cycas revoluta*	1	

*Dracaena marginata*	3	

*Elaeis guineensis*	4	

*Linum usitatissimum*	1	1

*Musa acuminata*	1	

*Nuphar advena*	2	

*Oryza sativa*	1	

*Persea americana*	2	

*Physcomitrella patens*	1	

*Pinus lambertina*	1	

*Pinus monticola*	5	

*Pinus taeda*	2	

*Sansevieria trifasciata*	2	

*Solanum lycopersicum*	2	3

*Spathiphyllum*	3	

*Triticum*-*Thinopyrum*	1	

*Zingiber cernuum*	1	

*Zingiber officinale*	2	

Total	53	10

Number of sequences from each plant species used in phylogenetic analysis. The number from each species used in the Pfam seed sequence is shown for comparison.

## Discussion

Previous studies of plant NBS-LRR sequences have suggested that only non-TIR-NBS-LRR sequences are present in monocots [7-9,13], and the sequences from the *Triticum-Thinopyrum *addition line [14] have not been mentioned in later studies [17,18,22,23]. A study in *Agrostis *species [22] reported two TIR-NBS-LRR sequences (Genbank: EE284250, EE284257). However, when these cDNA sequences are translated, they do not contain an open reading frame consistent with a NBS domain (data not shown), so it is unclear that these represent monocot TIR-NBS-LRR genes.

### Our PCR strategy amplified TIR-NBS-LRR sequences from dicot and gymnosperm DNA, but not from monocot DNA

In spite of attempts to bias amplification and cloning toward TIR-type NBS sequences, we did not find TIR-NBS-LRR sequences in any of the monocots we tested, although we easily cloned and sequenced TIR-type sequences from a gymnosperm, eudicot, and *Arabidopsis *control reactions. Although we expected to find the TIR class in dicots, a previous study in coffee did not report any TIR-type sequences [24].

Our results support the hypothesis that TIR-type NBS sequences are rare in monocots. We used diverse monocot taxa, including a species closely related to grasses (*C. blanda*) and a species from a basal monocot order (*Spathiphyllum *sp.). As with any PCR study, we cannot eliminate the possibility that TIR-NBS-LRR sequences in monocots are too divergent for our primers to amplify. Species-specific amplification has been reported [15] and more comparative work is needed to confirm that there are definitive consensus sequences for these motifs that are well-conserved across diverse taxa.

### Database searches and phylogenetic analysis show that TIR-type NBS sequences are present in basal angiosperms, but are rare in monocots and magnoliids

Using Pfam and GenBank, we obtained NBS sequences from monocots, a magnoliid, basal angiosperms, gymnosperms, and a bryophyte. The pine and moss sequences had been classified previously [10-12] and provided diverse lineages for comparison to the predominantly monocot and dicot sequences in our study. Based on the kinase-2 motif, TIR-type sequences were absent from monocots and magnoliids (with the exception of the reported *Triticum*-*Thinopyrum *sequences), but were present in basal angiosperms, gymnosperms, and bryophytes (Figure 2).

Our phylogenetic analysis (Figure 3) was consistent with previous analyses that showed a single TIR clade and multiple non-TIR clades [8,9]. The full NBS domain was not available for some sequences used in the analysis. We expect that the phylogenetic relationships will be clarified as more sequence becomes available. Our clear non-TIR clades corresponded to N3 and N4 (Cannon), with N1 and N2 split into several poorly-supported clades. Cannon's N4 clade did not include monocots, while our analysis placed a *Z. officinale *sequence in this clade (Figure 3). Cannon reported that N1.2 might be monocot specific, but our corresponding clade was not well-supported. Based on the current analysis, N3.2 may be monocot specific.

We expected to find both dicots and gymnosperms represented across TIR and non-TIR clades, but gymnosperm sequences were only found in the TIR and N4 clades. Both magnoliid sequences were non-TIR type sequences, and all basal angiosperm sequences were in the TIR clade (Figure 3). As more basal angiosperm sequences become available, we expect to also find non-TIR-type NBS sequences. Our results suggest that TIR-type sequences are rare in both magnoliids and monocots.

### TIR-NBS-LRR vs. the TIR domain

The rarity of TIR-NBS-LRR sequences in monocots does not necessarily reflect on the abundance of the TIR domain itself. Two similar protein families that may act as adapters (TIR-NBS and TIR-X) are found in monocots, but in low numbers compared to dicots and gymnosperms [25]. Many sequences in the databases are fragments, and some predicted non-TIR-NBS-LRR proteins may be members of these new families [25].

Classification of NBS-LRR proteins into TIR and non-TIR (Figure 1) is based on consensus motifs within the NBS domain (Table 1). Although these are assumed to be diagnostic [8], the presence or absence of a TIR domain has not usually been confirmed. We cannot eliminate the possibility that these motifs are not diagnostic. Further sequencing of the N-terminal regions of these genes is needed to confirm that our categorization is correct.

## Conclusion

We were unable to find monocot TIR-NBS-LRR sequences by PCR amplification or database searches. In contrast to previous studies, our results represent five monocot orders (Poales, Zingiberales, Arecales, Asparagales, and Alismatales) as well as basal angiosperms and magnoliids. Establishing the presence of TIR-type NBS sequences in basal angiosperms fills a gap in our knowledge of these important genes. Our results suggest that although TIR-type NBS-LRR sequences were present in early land plants, they have been reduced significantly in monocots. The sequences from the *Triticum-Thinopyrum *line [14] remain the only reported monocot TIR-NBS-LRR sequences. We do not know when these sequences were lost, but the *P. americana *sequences suggest that TIR-NBS-LRR sequences are rare in magnoliids as well. It is not clear whether these sequences were lost independently in both lineages or prior to their divergence. Further sequencing from additional taxa and confirmation that the motifs in the NBS region are diagnostic will be helpful in clarifying the evolutionary history of plant *R *genes.

## Methods

### Plants

We vegetatively propagated *Carex blanda *individuals collected from Kansas [26] and grew *Arabidopsis thaliana *from seeds (Ruth Shaw, University of Minnesota) in the greenhouse. *Draceana marginata*, *Sansevieria trifasciata*, *Spathiphyllum *sp., *Cycas revoluta*, and *Coffea canephora *came from the University of Kansas greenhouse.

### Genomic DNA isolation

We isolated genomic DNA from frozen plant material by grinding with a mortar and pestle in extraction buffer (0.2 M Tris-HCl pH 7.5, 0.25 M NaCl, 25 mM EDTA pH 8.0, 0.5% SDS) and incubating at 65°C for 10 minutes. We performed three extractions with phenol:chloroform:isoamyl alcohol (25:24:1); the final extraction used a phase-lock tube (Eppendorf). We precipitated the DNA and resuspended it in 100 μL10 mM Tris pH 8.0 with 10 μg RNaseA.

### Degenerate PCR

**Figure 4:**
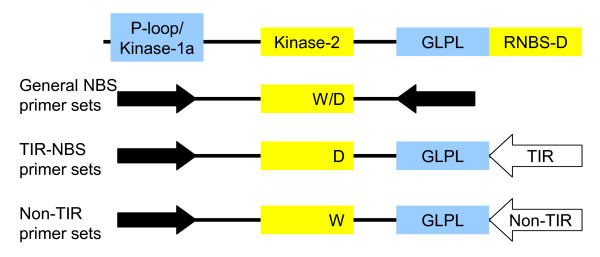
**Primer design**. The diagram shows the NBS domain motifs used in primer design. The motifs shown in blue are relatively conserved between TIR and non-TIR classes of NBS sequence while the domains in yellow have consistent differences. The three types of primer sets are shown with arrows to mark the location of the primers used. We used a total of 24 primer combinations that would specifically amplify TIR-NBS and non-TIR-NBS sequences, as well as combinations that would amplify all NBS sequences. All combinations were designed to amplify the kinase-2 region containing either a tryptophan (non-TIR) or aspartic acid (TIR) to aid in classification of the sequence.

We amplified 200 ng of genomic DNA with degenerate primers (Invitrogen). We used two 5' primers (P-loop/Kinase-1a region) and twelve 3' primers (GLPL and RNBS-D regions; Figure 4 and Table 4). The 50 μL reactions contained 2.5 units Platinum Taq polymerase (Invitrogen), 5 μL 10× buffer, 1.5 μL 50 mM MgCl_2_, 1 μL 10 mM dNTP mix, and 5 μL each degenerate primer (10 μM). The cycling parameters included initial denaturation at 94°C for 2 minutes, 35 cycles of 94°C for 30 seconds, 50°C for 30 seconds, 72°C for 1 minute, and a final extension at 72°C for 10 minutes.

**Table 4:** Primers used for amplification of NBS sequences

**Specificity (abbreviation)**	**Amino acid sequence**	**Degenerate primer (5'-3')**
Conserved P-loop/Kinase-1a (V)	GVGKTT	GGIGTIGGIAARACIAC

P-loop/Kinase-1a TIR (I)	GIGKTT	GGIATHGGIAARACIAC

RNBS-D TIR primer 1L (T1L)	FLHIACFF	RAARAARCAIGCDATRTGIARRAA

RNBS-D TIR primer 1 (T1)	FLHIAC	CAIGCDATRTGIARRAA

RNBS-D TIR primer 2 (T2)	FLHIAC	CANGCDATRTGAARRAA

RNBS-D TIR primer 3 (T3)	FLHIAC	CANGCDATRTGCARRAA

RNBS-D TIR primer 4 (T4)	FLHIAC	CANGCDATRTGGARRAA

RNBS-D TIR primer 5 (T5)	FLHIAC	CANGCDATRTGTARRAA

RNBS-D non-TIR primer 1 (nT1)	CFLYCALFPED	CYTCIGGRAAIARIGCRCARTAIARRAARC

RNBS-D non-TIR primer 2 (nT2)	CALFPED	CYTCNGGRAANARNGCACA

RNBS-D non-TIR primer 3 (nT3)	CALFPED	CYTCNGGRAANARNGCGCA

Conserved GLPL primer 1 (1)	CGGLPLA	GCIARIGGIARICCICCRCA

Conserved GLPL primer 2 (2)	CGGLPLA	GCNARNGGNARNCCNCCACA

Conserved GLPL primer 3 (3)	CGGLPLA	GCNARNGGNARNCCNCCGCA

Primers are based on previously reported consensus amino acid sequences [7], with the exception of the primer to the P-loop sequence GIGKTT, which was reported as the P-loop consensus sequence for the TIR-NBS-LRR group [9].

### Cloning and sequencing

We purified PCR products from agarose gels using the QIAquick gel extraction kit (QIAgen), ligated into the pCR4-TOPO vector (Invitrogen), and transformed into maximum efficiency DH5α competent cells. We isolated plasmid DNA from overnight cultures using a standard alkaline lysis protocol, digested with EcoRI, and sequenced representative clones with standard primers (T3/T7) at the University of Kansas sequencing facility or ACGT, Inc. We typically sequenced between one and three clones from each fragment. Sequences were submitted to GenBank with accession numbers EF687860-EF687864 and EF687876-EF687894.

### Database searches

We retrieved monocot (excluding Poales), gymnosperm, and bryophyte sequences from Pfam by viewing the NB-ARC domain (PF00931) species distribution. We retrieved sequences from GenBank by searching with known plant NBS sequences [see Additional File 2] and using the taxonomy reports to identify non-dicot, non-grass plant sequences with significant similarity (e < 0.05). We used EditSeq (Lasergene) for all sequence viewing and editing, and excluded sequences with internal stops or that lacked the diagnostic kinase-2 region.

### Phylogenetic analysis

We aligned sequences using the ClustalW algorithm in MegAlign (Lasergene) with manual adjustments as necessary. The alignment contained a core region of approximately 170 amino acids. We generated a scaffold tree based on the Pfam seed alignment and previous analyses [see Additional File 3] [8]. Phylogenetic analysis with parsimony criteria was performed using PAUP* 4.0 beta10 [27].

## Competing interests

The authors declare that they have no competing interests.

## Authors' contributions

DT participated in sample collection, carried out the PCR study, performed the database searches and phylogenetic analysis, and drafted the manuscript. HA participated in sample collection, study design, and manuscript revision. Both authors read and approved the final manuscript.

## Supplementary Material

Additional file 1 **Accession numbers for sequences used in the phylogenetic analysis that were obtained by PCR**. Accession numbers for sequences used to generate the phylogenetic tree shown in Figure 3 obtained by PCR. We chose the representatives shown by eliminating redundant sequences within a species (>70% identity). Click here for file

Additional file 2 **Accession numbers for sequences used in the phylogenetic analysis that were retrieved from online databases**. Accession numbers for sequences used to generate the phylogenetic tree shown in Figure 3 obtained from databases (Pfam or GenBank). Pfam 22.0 identified over 4000 plant sequences that contain the NB-ARC domain (PF00931). Of the 1215 monocot sequences, 1201 were from the grass family (Poaceae). The other 14 monocot sequences were from *Elaeis guineensis *(Arecales) and *Musa acuminata *(Zingiberales). We used both TIR and non-TIR sequences as queries in BLASTP and TBLASTN searches of the GenBank EST database. We increased the number of maximum targets to 1000 and performed independent searches with the organism set limited to plants, monocots, magnoliids, and basal angiosperms. The TIR-type NBS query sequences were Q42054_LINUS (Pfam), Q8LPB9_PHYPA (Pfam), Q6WE87_PINMO (Pfam), EF687876 (Genbank), and EF687894 (Genbank). The non-TIR-type query sequences were all from Genbank and included EF687875, EF687871, EF687860, EF687880, EF687878, BAB08632, and AU084895. We obtained the representatives shown by eliminating redundant sequences within a species (>70% identity). Click here for file

Additional file 3 **Accession numbers for sequences used to generate the scaffold tree**. Accession numbers for the sequences in Figure 3 that were used to make the scaffold tree. The Pfam seed alignment contains twelve amino acid sequences that represent the NB-ARC (NBS) domain, including ten from plants, one from *C. elegans *(CED-4), and one from humans (APAF-1). We compared these twelve sequences to a previous phylogenetic study of NBS sequences in plants that identified one TIR group and several non-TIR groups [8] to confirm that all identified subgroups were represented by sequences in the Pfam seed alignment. While some of the accession numbers for sequences used in the study corresponded to records that have been removed, we retrieved 79 sequences and aligned them with the 12 sequences from Pfam. Phylogenetic analysis using parsimony criteria generated a tree similar to that reported by Cannon (not shown). The ten seed sequences from plants represented four of the groups previously identified [8]: TIR, N1.1 N2, and N3. We added the four sequences from Cannon's analysis that represented N1.2 and N4 to the ten plant sequences. We eliminated the *C. elegans *sequence from the alignment because it contained a long insertion that was difficult to align. We also excluded O24015 from *Solanum lycopersicum *because it clustered with O24016 with 100% bootstrap support in all analyses. We kept human APAF-1 as an outgroup sequence. Click here for file

## References

[B1] Chisholm ST, Coaker G, Day B, Staskawicz BJ (2006). Host-microbe interactions: shaping the evolution of the plant immune response. Cell.

[B2] Jones JDG, Dangl JL (2006). The plant immune system. Nature.

[B3] Hammond-Kosack KE, Jones JDG (1997). Plant disease resistance genes. Annu Rev Plant Physiol Plant Mol Biol.

[B4] Biezen EA van der, Jones JDG (1998). The NB-ARC domain: a novel signalling motif shared by plant resistance gene products and regulators of cell death in animals. Curr Biol.

[B5] Dangl JL, Jones JDG (2001). Plant pathogens and integrated defence responses to infection. Nature.

[B6] Hulbert SH, Webb CA, Smith SM, Sun Q (2001). Resistance gene complexes: evolution and utilization. Annu Rev Phytopathol.

[B7] Meyers BC, Dickerman AW, Michelmore RW, Sivaramakrishnan S, Sobral BW, Young ND (1999). Plant disease resistance genes encode members of an ancient and diverse protein family within the nucleotide-binding superfamily. Plant J.

[B8] Cannon SB, Zhu H, Baumgarten AM, Spangler R, May G, Cook DR, Young ND (2002). Diversity, distribution, and ancient taxonomic relationships within the TIR and non-TIR NBS-LRR resistance gene subfamilies. J Mol Evol.

[B9] Bai J, Pennill LA, Ning J, Lee SW, Ramalingam J, Webb CA, Zhao B, Sun Q, Nelson JC, Leach JE, Hulbert SH (2002). Diversity in nucleotide binding site--leucine-rich repeat genes in cereals. Genome Res.

[B10] Akita M, Valkonen JP (2002). A novel gene family in moss (*Physcomitrella patens*) shows sequence homology and a phylogenetic relationship with the TIR-NBS class of plant disease resistance genes. J Mol Evol.

[B11] Liu JJ, Ekramoddoullah AK (2003). Isolation, genetic variation and expression of TIR-NBS-LRR resistance gene analogs from western white pine (*Pinus monticola *Dougl. ex. D. Don.). Mol Genet Genomics.

[B12] Jermstad K, Sheppard L, Kinloch B, Delfino-Mix A, Ersoz E, Krutovsky K, Neale D (2006). Isolation of a full-length CC-NBS-LRR resistance gene analog candidate from sugar pine showing low nucleotide diversity. Tree Genet Genomes.

[B13] Pan Q, Wendel J, Fluhr R (2000). Divergent evolution of plant NBS-LRR resistance gene homologues in dicot and cereal genomes. J Mol Evol.

[B14] Jiang S-M, Hu J, Yin W-B, Chen Y-H, Wang RR-C, Hu Z-M (2005). Cloning of resistance gene analogs located on the alien chromosome in an addition line of wheat-*Thinopyrum intermedium*. Theor Appl Genet.

[B15] Aswati Nair R, Thomas G (2007). Isolation, characterization and expression studies of resistance gene candidates (RGCs) from *Zingiber *spp. Theor Appl Genet.

[B16] Azhar M, Heslop-Harrison J (2008). Genomes, diversity and resistance gene analogues in *Musa *species. Cytogenet Genome Res.

[B17] Miller RN, Bertioli DJ, Baurens FC, Santos CM, Alves PC, Martins NF, Togawa RC, Souza MT, Pappas GJ (2008). Analysis of non-TIR NBS-LRR resistance gene analogs is *Musa acuminata *Colla: Isolation, RFLP marker development, and physical mapping. BMC Plant Biol.

[B18] Pei X, Li S, Jiang Y, Zhang Y, Wang Z, Jia S (2007). Isolation, characterization, and phylogenetic analysis of the resistance gene analogues (RGAs) in banana (*Musa *spp.). Plant Sci.

[B19] Angiosperm Phylogeny Group (2003). An update of the Angiosperm Phylogeny Group classification for the orders and families of flowering plants: APG II. Bot J Linn Soc.

[B20] Finn RD, Mistry J, Schuster-Bockler B, Griffiths-Jones S, Hollich V, Lassmann T, Moxon S, Marshall M, Khanna A, Durbin R (2006). Pfam: clans, web tools and services. Nucleic Acids Res.

[B21] Benson DA, Karsch-Mizrachi I, Lipman DJ, Ostell J, Wheeler DL (2007). GenBank. Nucleic Acids Res.

[B22] Budak H, Su S, Ergen N (2006). Revealing constitutively expressed resistance genes in *Agrostis species *using PCR-based motif-directed RNA fingerprinting. Genet Res.

[B23] Ameline-Torregrosa C, Wang B-B, O'Bleness MS, Deshpande S, Zhu H, Roe B, Young ND, Cannon SB (2008). Identification and characterization of nucleotide-binding-site-leucine-rich repeat genes in the model plant *Medicago truncatula*. Plant Physiol.

[B24] Noir S, Combes MC, Anthony F, Lashermes P (2001). Origin, diversity and evolution of NBS-type disease-resistance gene homologues in coffee trees (*Coffea *L.). Mol Genet Genomics.

[B25] Meyers BC, Morgante M, Michelmore RW (2002). TIR-X and TIR-NBS proteins:two new families related to disease resistance TIR-NBS-LRR proteins encoded in Arabidopsis and other plant genomes. Plant J.

[B26] Alexander H, Price S, Houser R, Finch D, Tourtellot M (2007). Is there a reduction in disease and pre-dispersal predation at the border of a host plant's range? Field and herbarium studies of *Carex blanda*. J Ecol.

[B27] Swofford D (1998). PAUP* Phylogenetic Analysis Using Parsimony (*and Other Methods) Version 4.

[B28] Moffett P, Farnham G, Peart J, Baulcombe DC (2002). Interaction between domains of a plant NBS-LRR protein in disease resistance-related cell death. EMBO J.

[B29] Aarts N, Metz M, Holub E, Staskawicz BJ, Daniels MJ, Parker JE (1998). Different requirements for *EDS1 *and *NDR1 *by disease resistance genes define at least two *R *gene-mediated signaling pathways in *Arabidopsis*. Proc Natl Acad Sci USA.

[B30] DeYoung BJ, Innes RW (2006). Plant NBS-LRR proteins in pathogen sensing and host defense. Nature Immunol.

