# Supplementary material for: TIR-NBS-LRR genes are rare in monocots: evidence from diverse monocot orders
Source: BMC Res Notes. 2009 Sep 28;2:197. doi: 10.1186/1756-0500-2-197 (PMC2763876; doi:10.1186/1756-0500-2-197)
Supplement: Additional file 1 — Accession numbers for sequences used in the phylogenetic analysis that were obtained by PCR. Accession numbers for sequences used to generate the phylogenetic tree shown in Figure 3 obtained by PCR. We chose the representatives shown by eliminating redundant sequences within a species (>70% identity). [file 1756-0500-2-197-S1.PDF]

Sequences amplified by PCR		
**Tree label**	**Species**	**Genbank Accession**
C. blanda A (TIR-)	*Carex blanda*	EF687860
C. blanda B (TIR-)	*Carex blanda*	EF687862
C. blanda C (TIR-)	*Carex blanda*	EF687871
C. blanda D (TIR-)	*Carex blanda*	EF687875
C. canephora A (TIR-)	*Coffea canephora*	EF687891
C. canephora B (TIR-)	*Coffea canephora*	EF687892
C. canephora C (TIR-)	*Coffea canephora*	EF687893
C. canephora D (TIR+)	*Coffea canephora*	EF687894
C. revoluta (TIR+)	*Cycas revoluta*	EF687876
D. marginata A (TIR-)	*Dracaena marginata*	EF687877
D. marginata B (TIR-)	*Dracaena marginata*	EF687878
D. marginata C (TIR-)	*Dracaena marginata*	EF687879
S. trifasciata A (TIR-)	*Sansevieria trifasciata*	EF687880
S. trifasciata B (TIR-)	*Sansevieria trifasciata*	EF687881
Spathiphyllum A (TIR-)	*Spathiphyllum sp.*	EF687882
Spathiphyllum B (TIR-)	*Spathiphyllum sp.*	EF687883
Spathiphyllum C (TIR-)	*Spathiphyllum sp.*	EF687890
