# Supplementary material for: TIR-NBS-LRR genes are rare in monocots: evidence from diverse monocot orders
Source: BMC Res Notes. 2009 Sep 28;2:197. doi: 10.1186/1756-0500-2-197 (PMC2763876; doi:10.1186/1756-0500-2-197)
Supplement: Additional file 2 — Accession numbers for sequences used in the phylogenetic analysis that were retrieved from online databases. Accession numbers for sequences used to generate the phylogenetic tree shown in Figure 3 obtained from databases (Pfam or GenBank). Pfam 22.0 identified over 4000 plant sequences that contain the NB-ARC domain (PF00931). Of the 1215 monocot sequences, 1201 were from the grass family (Poaceae). The other 14 monocot sequences were from Elaeis guineensis (Arecales) and Musa acuminata (Zingiberales). We used both TIR and non-TIR sequences as queries in BLASTP and TBLASTN searches of the GenBank EST database. We increased the number of maximum targets to 1000 and performed independent searches with the organism set limited to plants, monocots, magnoliids, and basal angiosperms. The TIR-type NBS query sequences were Q42054_LINUS (Pfam), Q8LPB9_PHYPA (Pfam), Q6WE87_PINMO (Pfam), EF687876 (Genbank), and EF687894 (Genbank). The non-TIR-type query sequences were all from Genbank and included EF687875, EF687871, EF687860, EF687880, EF687878, BAB08632, and AU084895. We obtained the representatives shown by eliminating redundant sequences within a species (>70% identity). [file 1756-0500-2-197-S2.PDF]

Additional sequences from Pfam		
**Tree label**	**Species**	**Accession number**
E. guineensis A (TIR-)	*Elaeis guineensis*	Q9SE13_ELAGV
E. guineensis B (TIR-)	*Elaeis guineensis*	Q9SE14_ELAGV
E. guineensis C (TIR-)	*Elaeis guineensis*	Q9SE15_ELAGV
E. guineensis D (TIR-)	*Elaeis guineensis*	Q9SE16_ELAGV
M. acuminata (TIR-)	*Musa acuminata*	Q8LJV6_MUSAC
P. patens (TIR+)	*Physcomitrella patens*	Q8LPB9_PHYPA
P. lambertiana (TIR-)	*Pinus lambertiana*	Q1L6F3_PINLA
P. monticola A (TIR+)	*Pinus monticola*	Q6WE82_PINMO
P. monticola B (TIR+)	*Pinus monticola*	Q6WE83_PINMO
P. monticola C (TIR+)	*Pinus monticola*	Q6WE84_PINMO
P. monticola D (TIR+)	*Pinus monticola*	Q6WE86_PINMO
P. monticola E (TIR+)	*Pinus monticola*	Q6WE87_PINMO
P. taeda A (TIR+)	*Pinus taeda*	Q8L8I7_PINTA
P. taeda B (TIR+)	*Pinus taeda*	Q8L8J1_PINTA
**Additional sequences from GenBank**		
**Tree label**	**Species**	**Accession number**
A. trichopoda (TIR+)	*Amborella trichopoda*	FD440429
N. advena A (TIR+)	*Nuphar advena*	CD475374
N. advena B (TIR+)	*Nuphar advena*	CV004886
P. americana A (TIR-)	*Persea americana*	CK756899
P. americana B (TIR-)	*Persea americana*	FD505444
Tri-Thi addition (TIR+)	*Triticum aestivum/ Thinopyrum intermedium alien addition line*	AAP03076
Z. cernuum (TIR-)	*Zingiber cernuum*	AY864999
Z. officinale A (TIR-)	*Zingiber officinale*	DY361741
Z. officinale B (TIR-)	*Zingiber officinale*	DY377843
