# Supplementary material for: TIR-NBS-LRR genes are rare in monocots: evidence from diverse monocot orders
Source: BMC Res Notes. 2009 Sep 28;2:197. doi: 10.1186/1756-0500-2-197 (PMC2763876; doi:10.1186/1756-0500-2-197)
Supplement: Additional file 3 — Accession numbers for sequences used to generate the scaffold tree. Accession numbers for the sequences in Figure 3 that were used to make the scaffold tree. The Pfam seed alignment contains twelve amino acid sequences that represent the NB-ARC (NBS) domain, including ten from plants, one from C. elegans (CED-4), and one from humans (APAF-1). We compared these twelve sequences to a previous phylogenetic study of NBS sequences in plants that identified one TIR group and several non-TIR groups [8] to confirm that all identified subgroups were represented by sequences in the Pfam seed alignment. While some of the accession numbers for sequences used in the study corresponded to records that have been removed, we retrieved 79 sequences and aligned them with the 12 sequences from Pfam. Phylogenetic analysis using parsimony criteria generated a tree similar to that reported by Cannon (not shown). The ten seed sequences from plants represented four of the groups previously identified [8]: TIR, N1.1 N2, and N3. We added the four sequences from Cannon's analysis that represented N1.2 and N4 to the ten plant sequences. We eliminated the C. elegans sequence from the alignment because it contained a long insertion that was difficult to align. We also excluded O24015 from Solanum lycopersicum because it clustered with O24016 with 100% bootstrap support in all analyses. We kept human APAF-1 as an outgroup sequence. [file 1756-0500-2-197-S3.PDF]

**Sequences used for scaffold tree**		
**Tree label**	**Species**	**Accession (P=Pfam, G=Genbank)**
A. thaliana A (TIR-)	*Arabidopsis thaliana*	DRL15_ARATH (P)
A. thaliana B (TIR-)	*Arabidopsis thaliana*	DRL24_ARATH (P)
A. thaliana C (TIR-)	*Arabidopsis thaliana*	R13L4_ARATH (P)
A. thaliana D (TIR-)	*Arabidopsis thaliana*	RP8L1_ARATH (P)
A. thaliana E (TIR-)	*Arabidopsis thaliana*	RPM1_ARATH (P)
A. thaliana F (TIR-)	*Arabidopsis thaliana*	RPS2_ARATH (P)
A. thaliana G (TIR-)	*Arabidopsis thaliana*	BAB08632 (G)
A. thaliana H (TIR-)	*Arabidopsis thaliana*	CAB86014 (G)
C. japonica (TIR-)	*Cryptomeria japonica*	AU084895 (G)
H. sapiens APAF (TIR-)	*Homo sapiens*	APAF_HUMAN (P)
L. usitatissimum (TIR+)	*Linum usitatissimum*	Q40254_LINUS (P)
O. sativa (TIR-)	*Oryza sativa*	AAG16860 (G)
S. lycopersicum A (TIR-)	*Solanum lycopersicum*	O24016_SOLLC (P)
S. lycopersicum B (TIR-)	*Solanum lycopersicum*	Q96485_SOLLC (P)
